# Aversion and attraction to harmful plant secondary compounds jointly shape the foraging ecology of a specialist herbivore

**DOI:** 10.1002/ece3.2082

**Published:** 2016-04-08

**Authors:** Parris T. Humphrey, Andrew D. Gloss, Nicolas M. Alexandre, Martha M. Villalobos, Marcella R. Fremgen, Simon C. Groen, Lisa N. Meihls, Georg Jander, Noah K. Whiteman

**Affiliations:** ^1^ Ecology and Evolutionary BiologyUniversity of ArizonaTucsonArizona85721; ^2^ Rocky Mountain Biological LaboratoryGothicColorado81224; ^3^ Boyce Thompson Institute for Plant ResearchIthacaNew York14853; ^4^ Present address: Organismic and Evolutionary BiologyHarvard UniversityCambridgeMassachusetts02138; ^5^ Present address: USDA‐ARS Plant Genetics Research UnitColumbiaMissouri65211; ^6^ Present address: Department of Integrative BiologyUniversity of CaliforniaBerkeleyCalifornia94720

**Keywords:** *Drosophila*, glucosinolate, inducible defense, jasmonic acid, oviposition, preference–performance

## Abstract

Most herbivorous insect species are restricted to a narrow taxonomic range of host plant species. Herbivore species that feed on mustard plants and their relatives in the Brassicales have evolved highly efficient detoxification mechanisms that actually prevent toxic mustard oils from forming in the bodies of the animals. However, these mechanisms likely were not present during the initial stages of specialization on mustard plants ~100 million years ago. The herbivorous fly *Scaptomyza nigrita* (Drosophilidae) is a specialist on a single mustard species, bittercress (*Cardamine cordifolia*; Brassicaceae) and is in a fly lineage that evolved to feed on mustards only in the past 10–20 million years. In contrast to many mustard specialists, *S. nigrita* does not prevent formation of toxic breakdown products (mustard oils) arising from glucosinolates (GLS), the primary defensive compounds in mustard plants. Therefore, it is an appealing model for dissecting the early stages of host specialization. Because mustard oils actually form in the bodies of *S. nigrita,* we hypothesized that in lieu of a specialized detoxification mechanism, *S. nigrita* may mitigate exposure to high GLS levels within plant tissues using behavioral avoidance. Here, we report that jasmonic acid (JA) treatment increased GLS biosynthesis in bittercress, repelled adult female flies, and reduced larval growth. *S. nigrita* larval damage also induced foliar GLS, especially in apical leaves, which correspondingly displayed the least *S. nigrita* damage in controlled feeding trials and field surveys. Paradoxically, flies preferred to feed and oviposit on GLS‐producing *Arabidopsis thaliana* despite larvae performing worse in these plants versus non‐GLS‐producing mutants. GLS may be feeding cues for *S. nigrita* despite their deterrent and defensive properties, which underscores the diverse relationship a mustard specialist has with its host when lacking a specialized means of mustard oil detoxification.

## Introduction

Many herbivorous insect lineages have evolved mechanisms allowing them to avoid or neutralize toxic plant defense compounds (Wittstock et al. 2004; Winde and Wittstock 2011). In some cases, these compounds are sequestered by insects and enhance resistance to predators and parasites, as in monarch butterflies (Brower and Glazier 1975; de Roode et al. 2011, 2013), pipevine swallowtails (Sime et al. 2000), and mustard‐feeding sawflies (Müller 2008). In herbivorous insect species that specialize on Brassicales plants, nearly all of those species that have been studied have highly derived mechanisms that allow disarming of the mustard oil bomb (Ratzka et al. 2002; Wittstock et al. 2004; Winde and Wittstock 2011). Specialized detoxification mechanisms are among the salient traits linked to ecological specialization on a narrow range of toxic host plants, as is typical for most herbivorous insect species (Ehrlich and Raven 1964; Mitter et al. 1988; Forister et al. 2015). But because such highly efficient mechanisms of resisting plant defenses evolved so long ago (on the order of hundreds of million years ago), it is difficult to establish whether they are indeed prerequisites for, or rather a consequence of, host plant specialization (Futuyma and Moreno 1988; Forister et al. 2015). Studies on taxa whose evolutionary transitions to herbivory and host specialization are relatively more recent may shed light onto the strategies employed by insects to overcome plant defensive chemistry. We tested the hypothesis that the relatively evolutionarily young mustard specialist *Scaptomyza nigrita* exhibits behavioral strategies that mitigate costs of ingesting mustard oils, given that it lacks a specialized means of avoiding exposure through detoxification (Gloss et al. 2014).

The dipteran leaf miners of the genus *Scaptomyza* (Drosophilidae) are herbivores whose diet breadth ranges from oligophagous to monophagous (Whiteman et al. 2011, 2012; Gloss et al. 2014). Current estimates place the origin of herbivory within this clade at ~10–20 MYA (Goldman‐Huertas et al. 2015). The monophagous *S. nigrita* is native to mountainous regions of western North America and occupies the alpine and sub‐alpine stream habitat of its sole host plant, *Cardamine cordifolia* (Brassicaceae; “bittercress”). Adult females of *S. nigrita* pierce the abaxial surface of leaves with dentate ovipositors and then feed on wound exudates prior to oviposition into a subset of the wounds. Larvae hatch and mine within the leaves through three instars until pupating within leaves. Defoliation by *S. nigrita* can reach up to 70% of leaf area by the end of a bittercress growing season (Collinge and Louda 1988). Community‐wide herbivory reduces fitness in bittercress (Louda 1984), and the contribution of *S. nigrita* to this effect is large.

This plant–insect system has served as a textbook example of how herbivores influence the distribution of a plant host (Louda and Rodman 1996; Ricklefs and Miller 2000). *S*.* nigrita* may achieve such high defoliating potential due to adaptive traits that mitigate toxicity of isothiocyanates, which are the hydrolysis products of glucosinolates (GLS), the primary defensive compounds in the Brassicales (Halkier and Gershenzon 2006). We recently found that *Scaptomyza* spp. herbivores, including *S. nigrita,* do not avert GLS breakdown into their toxic isothiocyanates (ITCs); instead, ITCs are processed in the body of these flies by a generalized detoxification pathway (Gloss et al. 2014), the same way as in generalist herbivores (Schramm et al. 2012). We therefore hypothesized that because these flies cannot biochemically avert GLS breakdown into ITCs, *S. nigrita* might minimize exposure to toxic mustard oils by avoiding GLS‐rich plant tissues behaviorally.

Consistent with this hypothesis, Humphrey et al. (2014) reported that less *S. nigrita* damage accumulated on bittercress plants in the field that had been experimentally treated with jasmonic acid (JA) versus mock‐treated control plants. JA is an endogenous plant hormone which positively regulates the induction of many anti‐herbivore defenses, including GLS (Halkier and Gershenzon 2006). This result established that the distribution of *S. nigrita* damage in a bittercress population is sensitive to local variation in anti‐herbivore defenses, consistent with earlier observational work showing that the distributional patterns of *S. nigrita* damage can be negatively associated with foliar GLS profiles in bittercress (Louda and Rodman 1983a,b), However, no experiments have been conducted that examine which aspects of *S. nigrita* foraging ecology, such as host choice or larval performance, are impacted by variation in plant defenses and contribute to the larger scale distributional patterns noted above.

Here, we combine observational work with laboratory and field experiments to determine how antiherbivore plant defenses (including GLS) affect the foraging ecology of the monophagous *S. nigrita*. We test whether, and under what conditions, antiherbivore defenses in bittercress serve as effective defenses, attractants, or deterrents to *S. nigrita* by utilizing several distinct means of manipulating foliar plant defenses in the foraging environment of adult and larval flies. First, we assayed *S. nigrita* adult choice and larval performance following JA treatments in bittercress transplanted from the field. We complemented this with direct measurement of foliar GLS induction in local and systemic leaves following JA treatment or *S. nigrita* larval feeding. We then made use of wild‐type (WT) and an isogenic GLS‐deficient knock‐out mutant (GKO) of *Arabidopsis thaliana* (Arabidopsis) to probe the causal role of GLS in the preference and performance of this insect on a model host plant in controlled laboratory choice trials (Whiteman et al. 2011, 2012).

Overall, our complementary experiments allow us to formulate a working hypothesis of how plant defensive chemistry shapes the foraging ecology of *S. nigrita* at the between‐patch, between‐plant, and between‐leaf levels. Further, we present experimental evidence indicating that biased leaf selection by female flies likely drives the distributional patterns of feeding damage in natural bittercress stands reported in earlier studies (Collinge and Louda 1988). We report several lines of evidence suggesting that *S. nigrita* adults avoid high concentrations of GLS, even though flies were more attracted to the GLS‐yielding Arabidopsis that harmed their larvae compared to GLS knock‐out Arabidopsis. These contrasting patterns underscore the important observation that inducible plant defensive chemicals may play multiple roles in the foraging ecology of a monophagous insect: as attractive host cues—similar to well‐known mustard specialists—yet also as deterrents, consistent with their role as effective direct defenses against *S. nigrita*.

Some mustard specialists may indeed “feed with impunity” (Wittstock et al. 2004) on their GLS‐producing host plants. We found, however, that specialized biochemical means of subverting plant secondary compounds such as those exhibited by these classic mustard specialists are not a precondition for the evolution of extreme host plant specialization of the type exhibited by *S. nigrita* (Gloss et al. 2014). Instead, this insect may rely to a large degree on the behavioral exploitation of “windows of opportunity” (Renwick 2002) during which antiherbivore defenses are relatively reduced.

## Materials and Methods

The field and laboratory experiments were conducted between 2009 and 2013 at the Rocky Mountain Biological Laboratory (RMBL) in Gothic, CO, and greenhouse work was conducted in 2010 at the University of Arizona, Tucson, AZ. Near the RMBL, bittercress is a self‐compatible out‐crosser, but also reproduces clonally via rhizomes and often occurs as patches with dozens of stems (ramets) for each genotype (genet) (Collinge and Louda 1988).

### Adult preference assays using *S. nigrita* and bittercress

In July 2011, we transplanted individual minimally damaged, nonflowering bittercress ramets from several neighboring patches from a single site (“Copper Creek site”, GPS coordinates N38 57.642, W106 58.421) into pots and held them under a 16:8 h light:dark cycle approximately 30 cm below 32W fluorescent bulbs in the laboratory at the RMBL for up to a week prior to treatment. Plants were potted in a 1:3:1 mix of vermiculite, Sunshine Mix #3, and fine sand, and received daily watering but were not fertilized. Prior to treatment, 10 groups of four plants were matched by stem height to reduce variance introduced by differences in plant size, which may influence host choice. Plants within each group were then randomized into pairs to either receive JA or a mock treatment solution. Using a blunt syringe (1 mL), two basal cauline leaves per stem were infiltrated with approximately 20 *μ*L of 1 mmol/L JA or sterile water (each in a solution of 0.42% methanol) as a control. Two days following treatment, the four plants in each group—two of each treatment—were randomized to positions within each of the 10 replicate mesh cages (35 × 35 × 35 cm, https://www.livemonarch.com/castle.htm) into which we released five adult female *S. nigrita* collected from the same site as the plants near the RMBL. After 24 h, we removed all flies and counted leaf punctures (“stipples”) created by the adult female flies. After correcting counts for any prior stipple damage from the field, stipple counts on treated leaves (“local”), remaining untreated leaves (“systemic”), and plant‐wide counts (“total”) were modeled separately with treatment and number of leaves on each stem as fixed effects, and cage number as a random effect, using negative binomial generalized linear mixed models (GLMMs) implemented in R v. 3.0.2 (R Core Development Team 2012) using package *lme4* (Bates et al. 2014). Coefficient estimates for fixed factors for this model and all other negative binomial GLMMs are presented as log rate ratios (LRRs) in tables and in their exponentiated form as rate ratios in the main text for ease of interpretation.

### Larval performance assays using *S. nigrita* and bittercress

In July 2010, we transplanted fifty‐five ramets from the field near the RMBL (“Site 401”, GPS coordinates N38 58.645, W106 59.392) into the laboratory (as above) and randomized them to receive treatment with either a solution of 1 mmol/L JA or a sterile water control (each in a solution of 0.2% ethanol). To apply treatments, we sprayed leaves until dripping using spray bottles. Treatments were applied once and then again 72 h later, after which we immediately transplanted a single field‐collected larva into the third‐lowest cauline leaf of each plant. We measured larval mass 24 h later using a fine balance (Sartorius). Because larvae were highly susceptible to desiccation during weighing, larvae used in this experiment were not weighed prior to implantation. Instead, larvae of the same size as the ones used in the experiment were weighed to determine larval mass at the start of the experiment as roughly 0.5 g. Larval mass was modeled as a function of treatment using a nonparametric Mann–Whitney *U*‐test implemented in R.

### JA and *S. nigrita* GLS induction experiments

Bittercress produces at least 12 GLS, including those that yield ITCs upon breakdown, as well as oxazolidinethione‐yielding GLS (Louda and Rodman 1983a,b; Rodman and Louda 1985). Rhizomes from a single clonal *C. cordifolia* plant were collected at the Copper Creek site in August 2010 and grown for 5 months in a greenhouse at the University of Arizona. Plants were grown under ambient light conditions in a 1:3:1 mix of vermiculite, Sunshine Mix #3, and fine sand. Plants were watered every 3 days and fertilized weekly (MiracleGro, Scotts Corporations, Marysville, OH). We randomized rosettes to be sprayed until dripping with either 1 mmol/L JA (in 0.42% methanol in deionized H_2_O) or a mock solution (0.42% methanol in deionized water). Treatments were applied at 0 h and again at 72 h. Two large (~3.5 cm^2^) and two small (~1.0 cm^2^) rosette leaves were collected from each of the five JA‐ and five mock‐treated plants 96 h after the initial treatment making sure the leaves were developmentally matched between treatments. We froze leaves at −80°C until they were shipped on dry ice for processing. Each pair of large or small leaf samples was pooled for each of the five plants per condition (20 data points in total), which were subsequently analyzed with HPLC for GLS profiles (see GLS detection below). Total and individual detected GLS concentrations (nmol per mg dry leaf tissue) in JA‐ or mock‐ treated leaves were compared using one‐way ANOVAs implemented in R. GLS data represent weighted averages of a small and large leaf from each plant.

In 2010, we transplanted 30 unmined and minimally stippled bittercress ramets from Site 401 and maintained them in the RMBL laboratory as in the above experiments. Plants were randomized into two groups, one of which received a single transplanted 2^nd^–3^rd^ instar larva collected from bittercress leaves at the same site into the third or fourth cauline leaf of each treated plant (*n* = 15), while the other (control) group received a pinprick at the base of the petiole to simulate transplantation (*n* = 15). Larvae were not initially weighed but instead were randomized across plants so that variation in initial larval mass was random with respect to plant treatment. Larvae mined inside the leaf into which they were implanted for 7 days, after which we harvested all leaves and created separate leaf tissue pools, from the oldest (lowest position 1) to youngest (highest position 7), by combining leaves from the 15 plants per treatment. This yielded 14 data points in total, each representing the pooled leaf tissue from 15 independent plants per condition. While this approach precludes estimation of variation within treatment–leaf position combinations, each data point nonetheless contains the total biological variation among 15 independent leaves sampled per treatment–position combination. Though sensitive to hidden outliers in the leaf pools, this approach on average yields data points where each is close to underlying mean values. We calculated log_2_‐fold differences in GLS content (both for individual GLS and total GLS) between *S. nigrita*‐infested versus mock‐treated plant tissues, both on a leaf‐by‐leaf and plant‐wide basis. Any technical error introduced during sample processing and GLS detection is common to leaf pools from both insect‐treated and mock‐treated sample pools. To test the null hypothesis that differences in individual GLS concentrations between leaf pools were random with respect to treatment, we used the nonparametric Wilcoxon ranked‐sum test (i.e., a sign test).

Additionally, we examined the relationship between foliar GLS and leaf position in noninduced (i.e., in mock‐treated) plants from the larval GLS induction experiment described above. Data for leaf position 8 was included in this analysis, whereas it was excluded in the previous experiment because all comparisons were made between developmentally matched leaf positions common to treatment and control plants. In addition, we included foliar GLS data (see GLS detection below) collected on leaf pools from positions 1–7 from unmined and minimally stippled plants that remained untreated but which were collected concurrently from Site 401. We modeled plant source (field vs. laboratory) and leaf position along the stem as predictors of nmol GLS per mg dry leaf tissue using a linear model implemented in R.

### GLS detection

GLS were detected by HPLC as desulfoGLS (Kim et al. 2004). Abundant desulfoGLS were identified by retention time, absorption spectra, mass spectra, and comparison to known Arabidopsis and *C. cordifolia* spectra. GLS quantities were calculated based on absorption and previously reported response factors (Brown et al. 2003). Detailed methods for GLS extraction and identification can be found in the Supporting Information as Appendix S1.

### Preference and performance assays using *S. nigrita* and Arabidopsis

In 2012, we grew presterilized Arabidopsis seeds in 42 × 42 mm rehydrated peat pellets (Novosel Enterprises, Oberlin, PA, USA) in flats under 32W lights (16:8 light:dark cycle) and watered them daily until plants were 4 weeks old. We used WT (Columbia‐0 [Col‐0]) Arabidopsis and an isogenic knockout mutant (GKO) [*cyp79b2 cyp79b3 myb28 myb29* in the Col‐0 background (Sun et al. 2009)] deficient in the production of both aliphatic and indolic GLS. GKO plants are also deficient in camalexin, owing to the dependence of its production on CYP79B2 and CYP79B3 (Glawischnig et al. 2004). Hereafter, we will refer to GKO plants as GLS‐deficient even though we recognize that they also lack camalexin.

Eighteen GKO and WT plants were randomly assigned to positions on a grid within each of two mesh cages (30 × 35 × 30 cm), into which we released 20 wild‐caught adult female flies collected at Site 401. After foraging in the mixed GKO+WT plant cages for 24 h, we removed the flies and counted total stipples made and eggs deposited in leaves of each plant. Into separate plants that were grown concurrently, we transplanted single wild‐collected *S. nigrita* larvae (also collected from bittercress leaves at Site 401) of uniform size (2^nd^–3^rd^ instar) into the largest developmentally matched leaves on GKO and WT plants (*n* = 36 per plant genotype). Developmentally matched leaves in Arabidopsis emerge at the same rosette position during plant growth. Larvae were not preweighed but were instead randomly assigned to plant genotype. This increases random variation within treatments but preserves our ability to appropriately test for mean differences in leaf area mined between plant genotypes. For each leaf in which the larva survived for 48 h, we removed and photographed the leaf, then traced leaf area mined using ImageJ (Abràmoff et al. 2004). Normality of model residuals was assessed using Shapiro─Wilk tests in R. Leaf area mined (mm^2^) was compared between plant genotypes using a one‐way ANOVA implemented in R. Leaf area mined is an informative proxy for performance of *Scaptomyza* spp. larvae on host plants (Whiteman et al. 2011).

### Examining within‐host leaf selection by *S. nigrita* in the field and laboratory

On 18 July, 2009, we conducted a survey of leaf miner damage on the leaves of 107 ramets randomly selected from a transect through bittercress patches along a stream at Site 401, which is the same survey site (“Site 2” of Collinge and Louda 1988, 1988) found to harbor bittercress ramets with *S. nigrita* damage enriched at lower leaf positions. In our survey, we counted the number of leaves on each of the sampled ramets and indicated whether each harbored a *S. nigrita* leaf mine. All leaves per stem were examined. To describe the distribution of leaf miner damage across stems, we fit a negative binomial distribution to the data with R package *fitdistrplus* (Delignette‐Muller and Dutang 2015) using maximum likelihood estimates of the dispersion parameter and mean. To test for an effect of leaf position on the probability of a leaf being mined, we used logistic regression with leaf position modeled as a continuous predictor of the probability of leaf miner damage.

We next experimentally assayed whether biased leaf selection by adult *S. nigrita* female flies resulted in a negative correlation between stipple damage and leaf position. In 2010, we collected undamaged bittercress ramets growing in open sun and under willow shade from Site 401 and planted them into a 1:3:1 mix of vermiculite, Sunshine Mix #3, and fine sand. Plants were watered daily but not fertilized, and were harbored under fluorescent lights four to a cage, having been randomized across eight replicate 30 × 35 × 61 cm mesh cages. Twenty‐four hours after plant transplantation to the laboratory, we released four adult female *S. nigrita* (collected from the same site as the plants) into each cage and allowed them to forage for 24 h. We then removed the flies and counted feeding punctures and eggs on each leaf using a dissecting microscope. We tested for an effect of leaf position on stipple and egg counts using GLMMs with negative binomial errors in R package *lme4* (Bates et al. 2014) with cage number (i.e., replicate) included as a random factor.

## Results

### *S. nigrita* choice and performance in relation to plant inducible defenses

**Figure 1:**
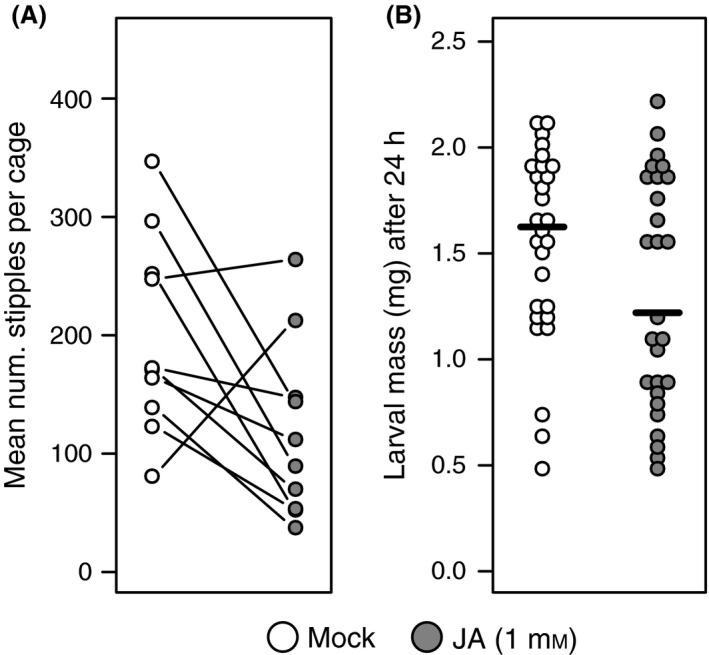
JA‐induced defenses shape *S. nigrita* adult preference (A) and larval performance (B) in laboratory assays using field‐collected bittercress. (A) Mean number of adult female feeding punctures (stipples) across plants in each condition for each cage (*n* = 10 cages total) compared using a GLMM (see Materials and Methods). Results displayed are for total stipples, rather than local or systemic stipples individually. (B) Larval mass gain after 24 h of feeding posttransfer in JA‐ (1 mmol/L) or mock‐treated leaves of field‐collected bittercress. See Table 1 for model result.

We conducted a laboratory choice experiment to test the hypothesis that JA‐dependent defenses deter foraging by adult *S. nigrita* females. In eight of 10 independent choice trials, adult *S. nigrita* female flies created fewer feeding punctures (“stipples”) in JA‐ versus mock‐treated bittercress plants: the expected plant‐wide stipple counts under a negative binomial GLM for JA‐treated plants were 58% of those for mock‐treated plants (Table 1; Fig. 1A). When considering only locally treated leaves, the reduction in damage following JA treatment was more pronounced compared to nontreated systemic leaves (Table 1).

**Table 1:** Results of negative binomial GLMMs for adult *S. nigrita* stipple counts in JA‐(1 mmol/L) versus mock‐treated bittercress

Leaf position(s)	Fixed factor	*ß* b (*±*SE)	*t*	*P*‐value
Local	Treatment (JA)	−0.73 (±0.23)	−3.2	<0.002
	Num. Leaves	0.03 (±0.03)	1.00	0.31
Systemic	Treatment (JA)	−0.47 (±0.26)	−1.82	0.07
	Num. Leaves	0.04 (±0.03)	1.28	0.20
Total^a^	Treatment (JA)	−0.57 (±0.22)	−2.58	<0.01
	Num. Leaves	0.03 (±0.03)	1.35	0.18

a Total represents plant‐wide stipples.

b Log rate ratios.

**Figure 2:**
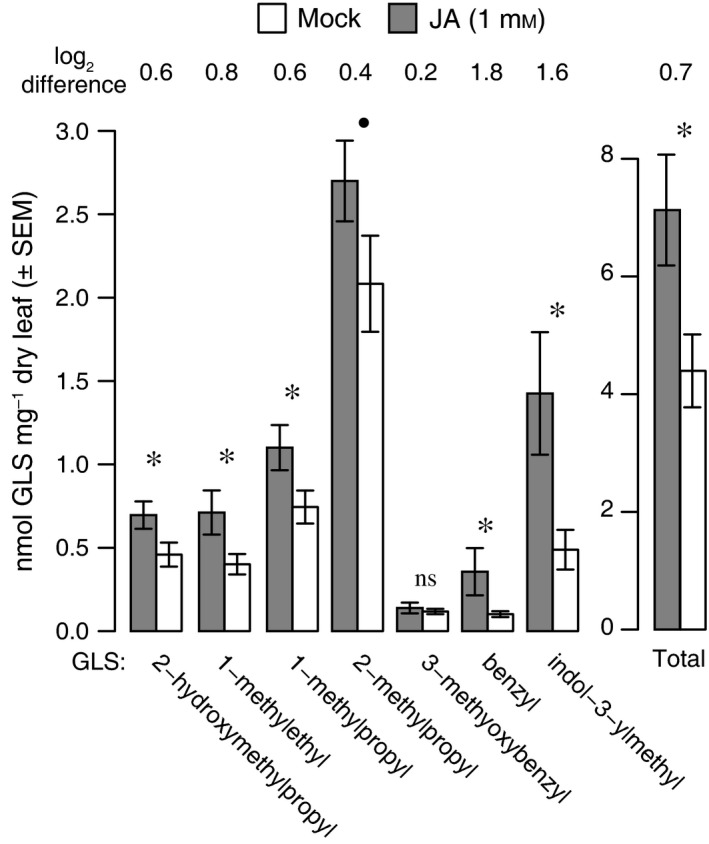
Foliar glucosinolate (GLS) induction in bittercress leaves following whole plant treatment with 1 mmol/L JA. Bars indicate absolute induction (nmol/mg leaf dry mass), while log_2_ differences indicate relative induction between JA and mock for each detected GLS. *P <* *0.05, •0.1 > *P *≥* *0.05, ns = nonsignificant (see Materials and Methods for statistical procedures).

We then tested if treatment of bittercress with JA increased resistance against *S. nigrita* and if JA treatment increased foliar GLS concentrations. In a larval transfer experiment, we found that larvae feeding for 24 h on JA‐pretreated plants weighed 15% less on average (mean mass of 1.31 mg ± 0.21 mg 95% CI), than those feeding on mock‐treated plants (1.55 mg ± 0.17 mg 95% CI; *P *=* *0.044, MWU one‐sided, Fig. 1B). Separately, plant‐wide treatment of bittercress rosettes with 1 mmol/L JA led to a concentration increase in five out of seven total detected GLS compared to mock‐treated leaves (Fig. 2). This constituted an overall 1.62‐fold increase of total GLS concentration (7.13 ± 1.84 95% CI vs. 4.40 ± 1.22 95% CI nmol/mg dry leaf mass, *P *<* *0.05; Fig. 2).

**Figure 3:**
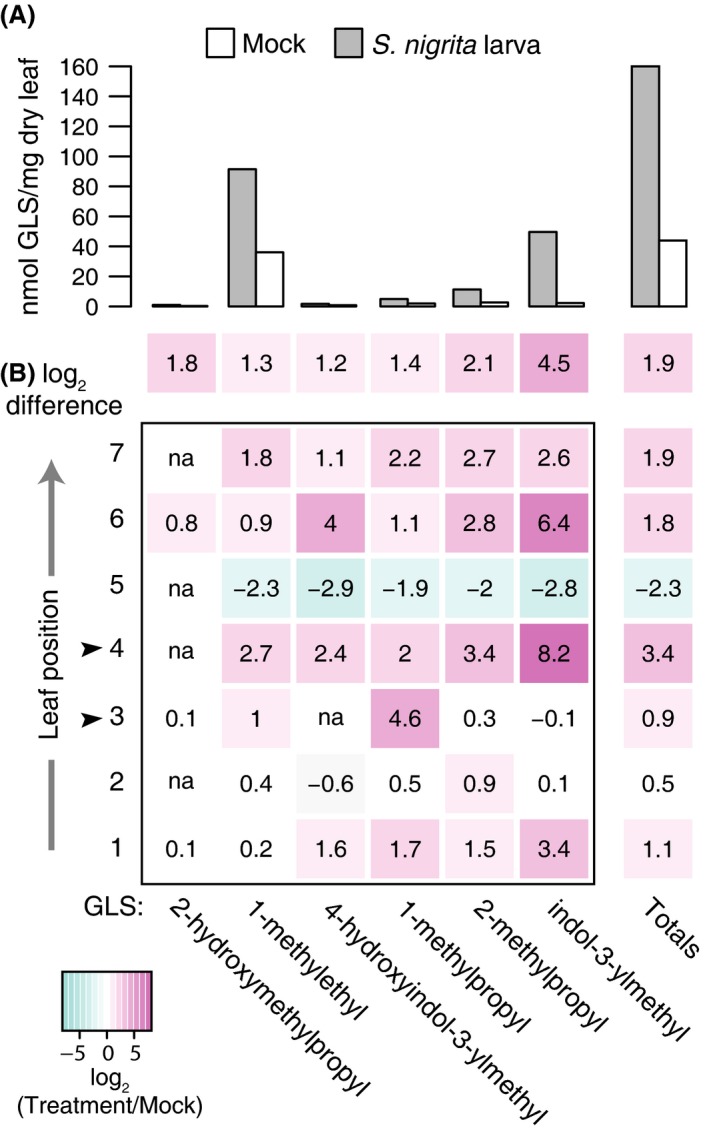
Individual and total glucosinolates (GLS) are induced across leaves in field‐collected bittercress stems 72 h post *S. nigrita* infestation. Data represent single measurements of pools of leaf discs from 15 leaves per leaf position per condition (*S. nigrita‐*infested vs. mock; see Materials and Methods). (A) Absolute GLS concentration (nmol/mg leaf dry mass) following *S. nigrita* implantation in bittercress leaf disc pools summed across leaf positions 1–7. (B) Relative GLS induction (log_2_ difference between treatment and mock) locally (implanted leaves; positions 3 and 4, indicated by arrows) and systemically in leaves along bittercress stem. Color key indicates magnitude of log_2_ difference between treatment and mock. “na” indicates none of the indicated GLS were detected in one or both of the leaf pools.

In parallel, we tested if leaf mining by *S. nigrita* larvae induced foliar GLS accumulation locally and systemically within bittercress ramets. *S. nigrita* larvae feeding in a basal leaf was sufficient to increase GLS concentration both locally (i.e., in damaged local leaves at positions 3 and 4, Fig. 3B) and in apical leaf positions (i.e., in undamaged and younger leaves at positions 6 and 7, Fig. 3B). All six detected GLS increased following *S. nigrita* treatment, (*P *=* *0.031, Wilcoxon signed‐rank test, two‐sided), constituting a 3.73‐fold net increase in total GLS concentration across all leaf positions (mock: 32.36 vs. *S. nigrita* larva: 145.01 nmol/mg dry leaf mass, Fig. 3A). Level of induction varied by type of GLS, with 1‐methylethyl‐GLS and indole‐3‐ylmethyl‐GLS increasing the most in apical leaves as well as plant‐wide (Fig. 3A). Although plant‐wide GLS concentration increased, leaf position 5 experienced a decrease in concentration of all individual foliar GLS (Fig. 3A) following *S. nigrita* damage. Leaf pools from each leaf positions from *S. nigrita*‐infested plants generally displayed an increase in GLS content versus mock‐treated leaf pools, with the highest inductions occurring in the position that received *S. nigrita* damage followed by the two most apical leaf positions.

Results from JA‐ versus *S. nigrita*‐induced changes in foliar GLS content are not directly comparable due to differences in sampling design and the plant life stages or genotypes (see Materials and Methods). Although the GLS detected in both assays largely overlapped, benzyl‐ and 3‐methoxybenzyl‐glucosinolate were only detected in JA‐induced rosette leaves, and 4‐hydroxyindol‐3‐ylmethylglucosinolate was only detected in *S. nigrita*‐infested cauline leaves.

### *S. nigrita* preference and performance in Arabidopsis

**Figure 4:**
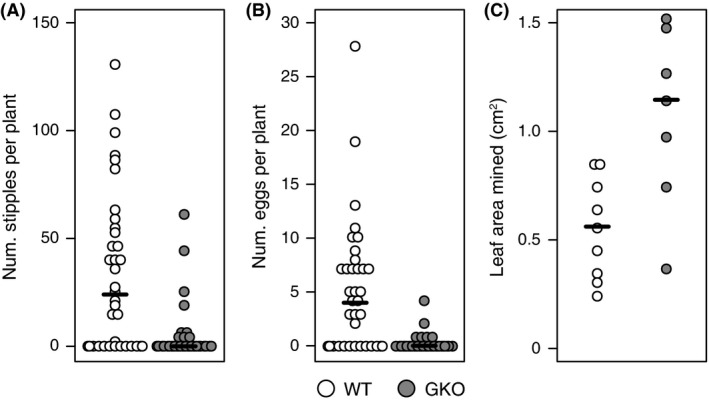
*S. nigrita* are attracted to, but develop more slowly on, *Arabidopsis thaliana* (Arabidopsis) with glucosinolates (GLS; WT) versus isogenic mutant plants without GLS (GKO). (A, B) Feeding (A) and oviposition (B) preference of *S. nigrita* adult females in cages with WT and GKO Arabidopsis. (C) Leaf area mined by transplanted *S. nigrita* larvae 48 h postimplantation in Arabidopsis with and without GLS. See Table 2 for statistical results.

We then tested whether *S. nigrita* females prefer host plants with or without foliar GLS, and monitored larval development in these plants, by conducting choice experiments with WT versus isogenic GLS‐deficient knockout (GKO; see Materials and methods for details on these genotypes) Arabidopsis plants. *S. nigrita* adult females made more stipples and laid more eggs on WT versus GKO Arabidopsis: the expected stipple counts under a negative binomial GLM for GKO plants were 28% of those for WT plants, while the expected egg counts on GKO plants were 6% of those for WT plants (Table 2; Fig. 4A and B). Although eggs hatched in both plant genotypes, no larvae successfully completed development.

**Table 2:** Model results for *S. nigrita* preference and performance on *A. thaliana* GKO versus WT

Response	Estimate (±SE)	Test statistic	*P*‐value
Stipples^a^	−1.26 (±0.42)	−3.04	0.0024
Eggs^a^	−2.83 (±0.42)	−6.8	<0.001
Leaf area mined (cm^2^)^b^	0.52 (±0.16)	3.18	0.0067

a GLMM with negative binomial errors. Estimate = log rate ratios; test statistic = *t*.

b One‐way ANOVA (i.e., two‐sided *t*‐test). Estimate = absolute effect size; test statistic = *t*.

Out of the 36 larvae per plant genotype that were transferred, only nine and seven survived after 48 h on WT and GKO plants, respectively. But the surviving larvae removed more leaf area if feeding on GKO compared to WT Arabidopsis plants (mean difference = 0.518 g, *P *<* *0.007; Table 2; Fig. 4C). When using a more conservative rank‐based test on these data, this difference of leaf area mined remained statistically significant (two‐sided Mann–Whitney *U*‐test, *P *=* *0.012).

### Within‐host foraging patterns of *S. nigrita*

**Figure 5:**
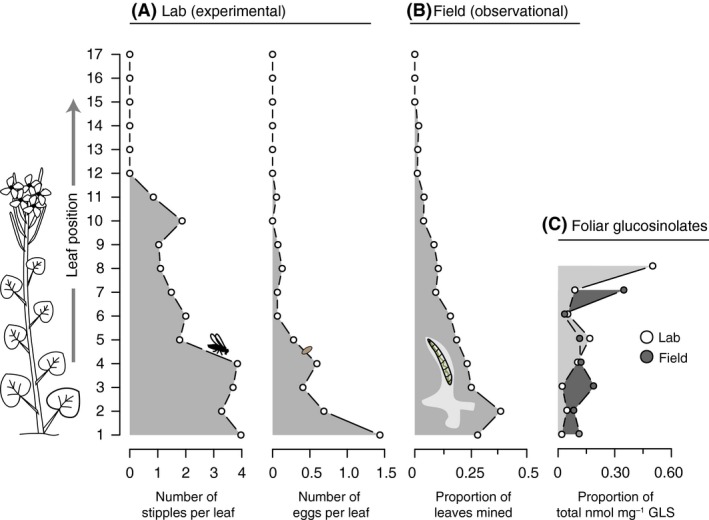
*S. nigrita* damage on bittercress stems is negatively correlated with leaf position and foliar glucosinolate (GLS) content. (A) Laboratory experiments demonstrated that the accrual of adult feeding damage (stipples) and egg deposition is highest on lower leaves during feeding trials. (B) Field observational data reveals that leaf miner damage is disproportionately higher on older leaves lower down on bittercress stems. Leaf position truncated at 17 leaves even though stems with up to 23 leaves were surveyed (none displayed mines). (C) Foliar GLS levels are highest in upper‐most (youngest) leaves in plants reared in the laboratory as well as similarly sized plants sampled from the field. Different heights of vertical axis in A–C reflect systematic differences in plant size. See Table 3 for statistical results.

Collinge and Louda (1988) noted that lower leaves on bittercress stems in the field tended to receive a disproportionately higher share of the damage by larval *S. nigrita*. We tested the repeatability of this pattern and found that, within the 107 bittercress stems surveyed in the field, the frequency of leaf damage by leaf miners steadily decreased with leaf position (Fig. 5B). Logistic regression revealed that the odds ratio (OR) of leaf mine damage was 0.75 (0.73–0.80 95% CI) between each pair of increasing leaf positions (i.e., decreasing leaf ages) along bittercress stems on average (Table 3; Fig. S2, Appendix S2). The overall probability of leaf miner damage was estimated as 0.38 (0.33–0.44 95% CI) on the lowest and oldest leaf position, which decreased to 0.003 (0.0017–0.0077 95% CI) by the youngest leaf position (position 20), according to model fits generated from logistic regression coefficient estimates (Table 3). Overall, the distribution of the number of mined leaves per stem closely followed a negative binomial distribution (mean = 1.88 ± 0.27 SE mined leaves per stem, dispersion parameter = 0.63 ± 0.14 SE; Fig. S1, Appendix S2).

**Table 3:** Model results for leaf position versus *S. nigrita* damage and foliar GLS

Data source	Response	Predictor	Estimate (±SE)	Test statistic	*P*‐value
Field (observational)^a^	Mines	Leaf position	−0.270 (±0.025)	−10.90	<0.0001
Lab (experimental)^b^	Stipples	Leaf position	−0.179 (±0.028)	−6.47	<0.0001
	Eggs	Leaf position	−0.403 (±0.055)	−7.31	<0.0001
Lab and Field^c^	nmol GLS/mg	Leaf position	2.65 (±1.09)	2.43	0.032
		Source	3.761 (±4.76)	0.79	0.444

a Logistic regression. Estimate = log odds ratio; test statistic = Wald's *z*.

b GLMMs with negative binomial errors. Estimate = log rate ratio; test statistic = *t*.

c Linear model. Estimate = slope; test statistic = *t*.

We then tested whether this within‐host pattern of damage arises from biases in the selection of leaves of different stem positions by foraging *S. nigrita* rather than from earlier and prolonged exposure of older lower leaves to foraging flies in the field. Experimental feeding trials with adult flies revealed a decreasing probability of both stipple and egg abundance with increasing bittercress leaf position (Table 3; Fig. 5A): the expected number of stipples and eggs, under negative binomial distributions, decreased by roughly 16% and 33% for each increasing leaf position, respectively. In contrast, we estimated a positive linear correlation between leaf position and the GLS content of independent sets of leaf pools from mock‐treated plants held under either field and laboratory conditions (Table 3; Fig. 5C), a pattern driven by the sharp increase in GLS concentration in the most apical leaf positions for each sample set. Analyzed another way, the probability of the most apical leaves containing the most extreme GLS concentrations under a null hypothesis, which assumes independence of leaf pools, is 0.018.

## Discussion

We found that the foraging ecology of *S. nigrita* is tightly linked to constitutive and JA‐dependent defenses of bittercress, but not in a manner that indicates a release from the cost of their ingestion, as expected from a monophagous species. Rather, the overall pattern of behavior is consistent with an herbivore whose ability to exploit its host is incomplete: their attraction to their host is tempered by the requirement to reduce exposure to the class of compounds that constrain larval development. Further, we found that these two processes—host attraction and aversion—may be mediated by the very same chemical class of compounds. Our studies of *S. nigrita* highlight the intricacies of the relationship that can arise relatively early on between an insect herbivore and its host plant following the evolution of host specialization.

Eliciting antiherbivore defenses in plants via JA treatments was sufficient to reduce feeding by adults in choice trials and reduce larval weight in feeding trials relative to mock‐treated plants (Fig. 1). Adult preference and larval performance measures were positively correlated and are consistent with expectations for a specialist whose larvae are restricted to feeding on the plant in which they were laid as eggs. The change in GLS profiles induced in bittercress leaves by *S. nigrita* larval damage largely overlapped with that induced by JA alone (Figs. 2, 3), indicating that foliar GLS are responsive to JA in bittercress. Thus, changes in foliar GLS content may at least contribute to the negative choice and performance outcomes of *S. nigrita* following JA treatment seen in this study.

In contrast, when utilizing an Arabidopsis mutant (GKO) deficient in the production of both aliphatic and indolic GLS as well as camalexin, *S. nigrita* adult preference was strongly in favor of WT plants that accumulated GLS (Fig. 4). Despite this preference, larvae implanted into WT Arabidopsis gained significantly less mass than the larvae in GKO mutant plants. Foliar GLS and/or camalexins can apparently function as attractants as well as defenses depending on the type of plants that foraging *S. nigrita* choose between.

The low larval survivorship in our experiments with WT and GKO Arabidopsis suggests that this plant is not a viable host for *S. nigrita*. But this does not preclude us from drawing some insight into *S. nigrita* foraging ecology on the basis of behavioral and performance differences *between* Arabidopsis genotypes. The value in these Arabidopsis experiments lies with how they may point to causal factors driving variation in foraging behavior. In this respect, our results indicate that the presence of GLS and/or camalexins *per se* in leaf tissue likely causes variation in *S. nigrita* behavior and performance. While subtle temperature‐dependent auxin deficiencies in Arabidopsis plants may result in developmental differences between GKO and WT Arabidopsis (Zhao 2010), previous metabolomics work with these genotypes has reported no other detectable systematic metabolic differences between these genotypes other than the predicted products of the four knocked‐out genes (Whiteman et al. 2012).

The preference of female flies for WT over GKO Arabidopsis suggests that their preference may depend on relative concentrations of GLS represented among the options being tested. Different behavioral responses may be triggered by the absence of GLS (as would be the case for a non‐Brassicaceae host plant) versus a higher or lower concentration of GLS relative to other leaves within the same foraging bout. Adult *Pieris* spp. and *Plutella xylostella* can depend on foliar GLS in order to accept a plant as a host (Renwick 2002; De Vos et al. 2008), and *S. nigrita* adults may similarly use GLS as a first stage in assessing host plant suitability.

Yet, the host choice hierarchy exhibited by *S. nigrita* at the between‐species level is not governed by simply the presence or absence of GLS as a whole. Experimental tests of host species selectivity conducted by Gloss et al. (2014) showed that *S. nigrita* adults strongly preferred feeding and ovipositing on bittercress even above other sympatric mustards in pairwise choice tests. That *S. nigrita* persists in monophagy despite the occurrence of other GLS‐yielding mustards sympatric with bittercress suggests that the presence of GLS in plant tissues may be necessary but not sufficient to promote usage of a given host species. The factors promoting extreme specialization on bittercress in this species have yet to be identified but may include the particular types of GLS present and their relative concentrations.

The patterns observed for *S. nigrita* feeding on Arabidopsis may be viewed as paradoxical in light of previous work with specialists of Brassicaceae. Using Arabidopsis knock‐out mutants deficient in either or both aliphatic and indolic glucosinolates, Müller et al. (2010) found that these classes of GLS additively promote oviposition by adult *Pieris rapae* and stimulate feeding by larvae of *P. rapae* and *Plutella xylostella*, and did not decrease larval or pupal performance measures. In contrast, the generalists (*Spodoptera exigua,* and *Trichoplusia ni*) and non‐crucifer‐specialist (*Manduca sexta*) also assayed each developed significantly faster on GLS knockout plants, indicating a cost to their performance of foliar GLS ingestion. Our results with *S. nigrita* mirror those for these known lepidopteran specialists where host choice is concerned. However, for larval performance, our results align more with the generalists whose larvae developed less well in GLS containing plants. One interpretation of this pattern for *S. nigrita* is simply that this species may require foliar GLS as a host acceptance cue prior to exhibiting feeding and oviposition search behavior along a negative GLS concentration gradient. The foraging patterns exhibited by *S. nigrita* may reflect a transitional strategy that may also help to reinforce a monophagous diet breadth. Additional experiments using Arabidopsis and bittercress with a well resolved gradient in foliar GLS may allow us to uncover an acceptance/avoidance GLS concentration threshold that relates to this apparent behavioral dichotomy.

These results complement those from prior work with *S. nigrita,* which together help to illustrate how the GLS content of a plant perceived by *S. nigrita* may interact with the spatial scale at which it is perceived. Exogenous JA treatment of sunny bittercress patches increased the local burden of leaf miner damage on bittercress leaves compared to control patches (Humphrey et al. 2014). However, at the within‐patch scale, paired mock‐treated stems experienced more damage compared to their neighboring JA‐treated stems. Attraction to JA‐induced host cues between patches, combined with aversion to JA treatment at between‐stem scales as found in this study, may underlie this pattern of associational susceptibility.

Both our observational and experimental studies with bittercress revealed that within‐plant feeding and oviposition choices by adults are strongly biased toward lower, older leaves. This choice bias is sufficient to explain the field‐scale observational pattern of increased incidence of larval damage on lower leaves found in our study and by Collinge and Louda (1988), indicating that leaf phenology alone does not explain the natural distribution of *S. nigrita* damage on bittercress stems. We also found that the youngest, apical bittercress leaves contained the highest concentrations of GLS when examined in untreated or mock‐treated bittercress leaves (Fig. 5C). A linear model likely does not capture the ways in which foliar GLS actually vary with leaf position; additional experiments are required to better describe the functional form of constitutive and inducible GLS variation along many individual bittercress stems. However, in addition to apical leaves being constitutively enriched for GLS, our study showed that apical leaves displayed a relatively greater GLS enrichment following distal *S. nigrita* larval damage compared to lower leaves (Fig. 3B). Thus, our study supports a model where apical leaves harbor the highest base‐line levels of foliar defenses against *S. nigrita* and become the most systemically induced in following *S. nigrita* larval damage. Both patterns are consistent with the avoidance response seen in adult *S. nigrita* choice tests and reduced larval weight gain following plant JA treatment. These results contrast with what is known about pierids, which can prefer plants (Renwick 2002) or tissues with relatively higher GLS content (Smallegange et al. 2007; Müller et al. 2010). But because additional factors confounded with GLS content such as leaf abscission rate (Bultman and Faeth 1986), nutrient density and/or leaf toughness can vary with leaf age (Collinge and Louda 1988; Travers‐Martin and Müller 2008), establishing the causal factors driving leaf choice within stems awaits future experiments.

Prior observational studies showed that foliar GLS content of bittercress in the field can vary widely (Louda and Rodman 1983a,b). Abiotic or other developmental factors that affect expression of JA‐regulated GLS accumulation are thus likely to also influence the probability and the extent of damage by *S. nigrita* in the field. Furthermore, we expect a large proportion of the standing variation in antiherbivore defenses, and thus GLS content, in the field also arises from prior biotic interactions that impart a lasting phenotypic effect on the plant (Poelman et al. 2008, 2011). In the foraging experiment of Humphrey et al. (2014), *S. nigrita* larval damage was elevated on plants that had been pretreated locally with salicylic acid (SA) in the field compared to neighboring mock‐treated plants, consistent with expectations that SA would diminish JA‐dependent defenses via SA–JA crosstalk (Koornneef and Pieterse 2008; Thaler et al. 2012). *Pseudomonas* spp. infection induces SA signaling and can promote insect damage locally and systemically in laboratory‐based studies (Cui et al. 2002, 2005; Chung et al. 2013; Groen et al. 2013), including for *Scaptomyza* spp. (Humphrey et al. 2014). We hypothesize that the preference of *S. nigrita* for plants treated with bacteria or SA may be driven by suppression of defenses that rely on JA induction, such as GLS enrichment.

## Conclusions

Some mustard specialists “feed with impunity” on GLS‐producing host plants (Wittstock et al. 2004). In contrast, we found that specialized biochemical means of plant secondary compound avoidance such as those exhibited by these classic mustard specialists are not a precondition for the evolution of extreme specialization on mustards (Gloss et al. 2014). *S. nigrita* may instead rely to a large degree on the behavioral exploitation of “windows of opportunity” (Renwick 2002) during which host defenses are relatively reduced. Nonetheless, strong attraction to GLS‐producing but not mutant Arabidopsis without GLS by *S. nigrita* reflects a host preference similar to other specialists (Goldman‐Huertas et al. 2015). Together, these results reveal that extreme specialization in *S. nigrita* does not require highly derived physiological mechanisms to overcome plant defenses. Rather, behavior may be the first phenotype to change, as proposed by Bernays and Chapman (1994).

## Data Accessibility

All data and R scripts will be deposited in the Dryad data repository (http://dx.doi.org/10.5061/dryad.rq436).

## Conflict of Interest

None declared.

## Supporting information


**Appendix S1.** Detailed glucosinolate extraction and identification procedures.
**Appendix S2.** Detailed results regarding the between‐ and within‐host distribution of damage by *S. nigrita* on bittercress in the field.
**Figure S1.** Negative binomial model fit for the distribution of mined leaves per stem in a systematic survey of bittercress stems in the field.
**Figure S2.** Observational data combined with logistic regression model fit of leaf position versus the probability a leaf at a given position was mined.
