# Supplementary material for: Aversion and attraction to harmful plant secondary compounds jointly shape the foraging ecology of a specialist herbivore
Source: Ecol Evol. 2016 Apr 8;6(10):3256–68. doi: 10.1002/ece3.2082 (PMC4829532; doi:10.1002/ece3.2082)
Supplement: Supplementary file 1 — Appendix S1. Detailed glucosinolate extraction and identification procedures. Appendix S2. Detailed results regarding the between‐ and within‐host distribution of damage by S. nigrita on bittercress in the field. Figure S1. Negative binomial model fit for the distribution of mined leaves per stem in a systematic survey of bittercress stems in the field. Figure S2. Observational data combined with logistic regression model fit of leaf position versus the probability a leaf at a given position was mined. [file ECE3-6-3256-s001.docx]

**Appendix S1: Detailed glucosinolate extraction and identification procedures.**

Leaf samples were lyophilized, dry weight was recorded, samples were placed in 2 mL microcentrifuge tubes containing two 3 mm steel ball bearings, and samples were crushed by shaking for 3 min on a Harbil model 5G-HD paint shaker. To each sample, 750 µL of 80% methanol and 5 µL of 5 mM sinigrin were then added. Samples were shaken again and heated at 75 °C for 15 min. After centrifugation at 10,000 x g for 10 min, 600 µL of each supernatant was added to 0.5 mL volume drip columns prepared with Sephadex A-25 (Amersham). Columns were washed twice with 1 mL methanol and twice with 1 mL dH_2_O. We then added 100 µL 15 mg/ml aryl sulfatase (Sigma, St. Louis, MO) to each column, which were then incubated overnight in the dark at 23 ºC. Samples containing desulfoGLS were eluted with 200 µL 80% methanol followed by 200 µL dH_2_O. Samples were evaporated in a Savant SC 110 rotary evaporator (Savant Instruments, Farmingdale, NY) and resuspended in 50 µL dH_2_O. DesulfoGLS were separated on a LiChrospher RP-18, 250 x 4.6 mm, 5 µm, column (Agilent, Santa Clara, CA) using a Waters 2695 HPLC and detected using a Waters 2996 photodiode array detector and a Micromass Quattro II mass spectrometer (Waters, Milford, MA). For HPLC separation, the mobile phases were A, water, and B, 90% acetonitrile, at a flow rate of 1.2 ml min^-1^ at 23 °C. Column linear gradients for samples were: 0–1 min, 98% A; 1–6 min 94% A; 6–8 min, 92% A; 8–16 min, 77% A; 16–20 min, 60% A; 20–25 min, 0% A; 25–27 min hold 0% A; 27–28 min, 98% A; 28–37 min, 98% A.

**Appendix S2. Detailed results regarding the between- and within-host distribution of damage by *S. nigrita* on bittercress in the field.**

******

***Figure S1.*** Negative binomial model fit for the distribution of mined leaves per stem in a systematic survey of bittercress stems in the field. Empirical = the shape of the distribution from the data. Theoretical = the shape of the distribution after estimating the negative binomial size shape parameters (see *Materials and Methods*, main text).

******

***Figure S2****.* Observational data combined with logistic regression model fit of leaf position vs. the probability a leaf at a given position was mined. Binary data points are jittered for visual purposes only. Model accounts for different numbers of leaves on stems (see *Materials and Methods* and Table 3, main text).
